# Long-Term Proton Pump Inhibitor Use and the Risk of Kidney Disease, Dementia, and Fractures: A Systematic Review

**DOI:** 10.7759/cureus.90627

**Published:** 2025-08-20

**Authors:** Mahrukh Chaudhry, Mussavir Elahi, Syed Hassan Ali Bukhari, David Ibikunle, Anil Koirala, Sara Zubair Ahmed, Mehak G Mastoi, Nimra Jabeen, Kirshan Lal, Ahmed Khan, Umer Ali

**Affiliations:** 1 Internal Medicine, King Edward Medical University, Lahore, PAK; 2 General Surgery, Sandeman Provincial Hospital, Quetta, PAK; 3 Internal Medicine, Redeemer’s Health Centre, Ibadan, NGA; 4 Gastroenterology and Hepatology, Obafemi Awolowo University Teaching Hospital, Ile-Ife, NGA; 5 Internal Medicine, Nepalgunj Medical College Teaching Hospital, Kohalpur, NPL; 6 Internal Medicine, Baqai Medical University, Karachi, PAK; 7 Geriatrics, Montefiore Medical Center Wakefield Campus, New York, USA; 8 Internal Medicine, Interfaith Medical Center, New York, USA; 9 Internal Medicine, HITEC Institute of Medical Sciences, Taxila, PAK; 10 Pediatrics, Shaheed Mohtarma Benazir Bhutto Medical University, Larkana, PAK; 11 Internal Medicine, Liaquat College of Medicine & Dentistry, Karachi, PAK; 12 Internal Medicine, Services Hospital, Lahore, PAK

**Keywords:** bone fractures, cardiovascular risk, chronic ppi use, cognitive decline, deprescribing, kidney disease, long-term adverse effects, observational studies, proton pump inhibitors, systematic review

## Abstract

Chronic use of proton pump inhibitors (PPIs), although effective for acid-related disorders, has raised concerns regarding their association with various long-term adverse health outcomes. This systematic review aimed to synthesize evidence from observational studies and meta-analyses evaluating the potential risks linked to prolonged PPI exposure, including cognitive decline, chronic kidney disease, bone fractures, and cardiovascular events. A comprehensive literature search was conducted using PubMed, Scopus, Web of Science, and Embase, focusing on studies involving adult human populations and reporting adjusted risk estimates. Eight studies met the inclusion criteria, encompassing prospective cohorts, cross-sectional analyses, and systematic reviews. A narrative synthesis approach was adopted due to the heterogeneity of study designs and outcome measures. While findings across studies varied, several demonstrated statistically significant associations between long-term PPI use and increased risks of adverse outcomes, particularly in elderly or comorbid populations. However, many studies were limited by confounding, bias, and the inability to establish causality. The evidence underscores the need for cautious and judicious prescribing of PPIs, with periodic reassessment of the indication and consideration of deprescribing in low-risk patients. Further high-quality prospective studies and randomized trials are warranted to clarify these associations and guide clinical decision-making.

## Introduction and background

Gastroesophageal reflux disease (GERD) and acid peptic disease (APD) are common gastrointestinal conditions affecting a significant proportion of the global population. These disorders often require long-term pharmacologic management, with acid-suppressive medications, such as proton pump inhibitors (PPIs), H2-receptor antagonists, and antacids, being the mainstay of treatment [1,2]. Among these, PPIs are particularly favored for their potent and sustained suppression of gastric acid secretion, making them highly effective in symptom control and mucosal healing. As a result, their use has expanded significantly over the past two decades, both for on-label indications and off-label prophylactic purposes in various clinical settings [3].

Despite their efficacy, concerns have emerged regarding the safety profile of long-term PPI and antacid use. Several observational and interventional studies have reported potential associations between chronic PPI use and a range of adverse outcomes, particularly chronic kidney disease (CKD), clinically confirmed cognitive disorders such as dementia (typically diagnosed through standardized clinical criteria, e.g., Diagnostic and Statistical Manual of Mental Disorders, Fifth Edition or equivalent), and an increased risk of radiologically verified bone fractures, often attributed to impaired calcium absorption [4]. The underlying mechanisms proposed for these associations include alterations in gut microbiota, nutrient malabsorption, vascular endothelial dysfunction, and cumulative toxic effects on renal tubular cells. While some studies have supported these associations, others have failed to demonstrate a definitive link, leading to ongoing debate and clinical uncertainty [5].

Given the widespread use of acid-suppressive therapy and the potentially serious consequences of long-term adverse outcomes, there is a growing need to critically examine the available evidence [6]. Previous reviews have often focused on isolated outcomes or lacked updated data. This underscores the importance of synthesizing high-quality, recent evidence to guide clinical decision-making and promote safe prescribing practices [7]. The objective of this systematic review is to evaluate and synthesize current evidence from meta-analyses, cohort studies, and other high-quality sources examining the association between long-term use of antacids, particularly PPIs, for GERD and APD, and the risk of developing CKD, dementia, and bone fractures.

## Review

Methodology

Study Design and Protocol

This systematic review was conducted in adherence to the Preferred Reporting Items for Systematic Reviews and Meta-Analyses (PRISMA) 2020 [8] guidelines to ensure methodological rigor and transparency. The research question was structured using the PICO framework [9], where the population consisted of adult human subjects exposed to long-term PPI use, the intervention was chronic use of PPIs, the comparison group included either non-users or users of alternative acid-suppressive therapies, and the outcomes assessed included adverse effects such as cognitive decline, kidney disease, and bone fractures. For this review, “long-term” or “chronic” PPI use was defined as regular use for more than three months. This threshold has been frequently used in prior gastroenterology and pharmacoepidemiological studies to distinguish short-term therapeutic courses from prolonged exposure, and therefore, provides a clinically relevant definition for evaluating potential adverse effects.

Eligibility Criteria and Search Strategy

Studies were included if they were peer-reviewed, published in the English language, involved human participants aged 18 years or older, and specifically examined the long-term effects of PPI use. Eligible designs included prospective cohort studies, cross-sectional studies, and systematic reviews and meta-analyses of observational data. We excluded narrative reviews, randomized controlled trials, editorials, letters, conference abstracts, pediatric studies, animal studies, and any research that failed to report adjusted effect estimates. Importantly, although data extraction included details on the “type and duration of antacid use,” this terminology referred exclusively to PPIs (omeprazole, esomeprazole, pantoprazole, lansoprazole, rabeprazole). No non-PPI antacids (such as calcium carbonate or H₂-receptor antagonists) were included, ensuring consistency with the study scope.

The literature search was performed using electronic databases such as PubMed, Scopus, and Embase. Boolean operators and Medical Subject Headings (MeSH) terms were employed to optimize retrieval. For example, the PubMed search strategy included: (“proton pump inhibitors”[MeSH Terms] OR “PPI” OR “proton pump inhibitor”) AND (“long-term use” OR “chronic use”) AND (“cognitive impairment” OR “dementia” OR “renal disease” OR “chronic kidney disease” OR “fractures” OR “adverse effects”). Reference lists of included articles were also manually screened to identify additional relevant studies.

Study Selection and Data Extraction

After duplicate records were removed, two independent reviewers screened titles and abstracts to identify potentially relevant studies. Full texts of selected articles were then assessed against the inclusion and exclusion criteria. Any disagreements regarding study eligibility were resolved through discussion and consensus. A structured data extraction sheet was used to collect key information from each study, including the first author’s name and year of publication, study type, population/sample size, type and duration of PPI use, primary outcomes assessed, main findings, effect estimates, and confounders adjusted. All data extraction steps were performed by two reviewers independently to ensure accuracy and minimize bias.

Quality Assessment and Risk of Bias

The methodological quality and risk of bias of each included study were evaluated using tools tailored to the specific study design. For prospective cohort studies, the Newcastle-Ottawa Scale (NOS) [10] was applied, which considers factors such as selection of participants, comparability of cohorts, and outcome assessment. Studies scoring 7 or more on the NOS were categorized as high quality. Cross-sectional studies were assessed using the AXIS (Appraisal Tool for Cross-Sectional Studies) tool [11], which examines design quality, potential for selection bias, clarity of objectives, and outcome reporting. For systematic reviews and meta-analyses of observational studies, the AMSTAR 2 [12] (A MeaSurement Tool to Assess systematic Reviews) checklist was employed, focusing on protocol registration, search strategy, study selection, risk of bias evaluation, and data synthesis. Each risk of bias judgment was independently performed by two reviewers, and discrepancies were resolved through mutual agreement after discussion.

Data Synthesis

Due to the heterogeneity in study designs, populations, outcome measures, and statistical methods across the included studies, a narrative synthesis approach was employed. The findings were grouped based on the nature of the study design, i.e., prospective cohort, cross-sectional, and systematic reviews/meta-analyses, and key themes related to chronic PPI use and its associated adverse outcomes were identified and summarized. Effect estimates such as odds ratios, hazard ratios, and relative risks were qualitatively compared where available, while attention was given to confounding variables adjusted for in each study. Patterns and consistencies across studies were highlighted to assess the strength of associations, while inconsistencies and methodological limitations were noted to guide interpretation. This approach allowed for a comprehensive understanding of the evidence base, despite variability in analytical techniques and outcome reporting.

Results

Study Selection Process

**Figure 1:**
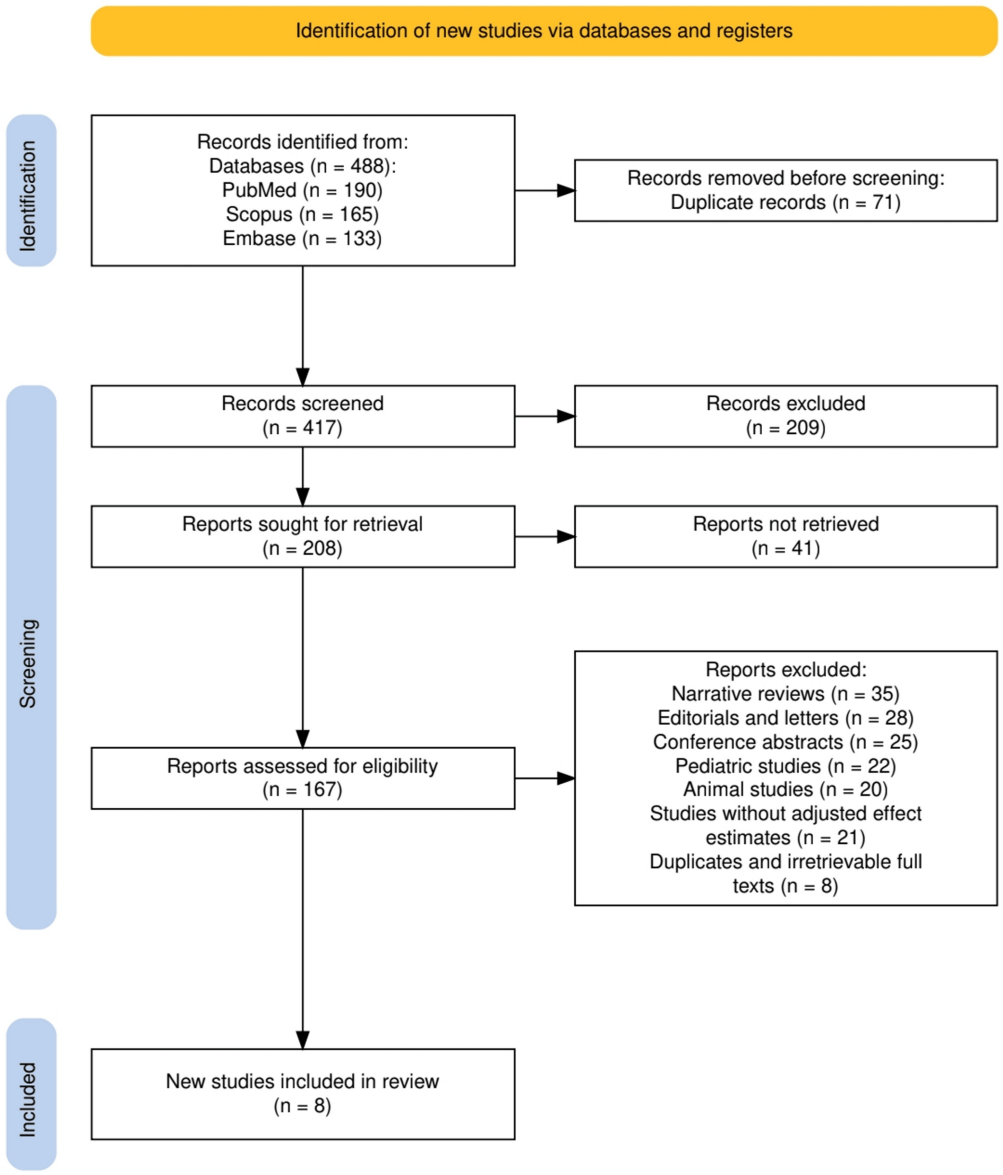
The PRISMA flow diagram showing the study selection process. PRISMA: Preferred Reporting Items for Systematic reviews and Meta-Analyses

The selection process for eligible studies is illustrated in Figure 1, which follows the PRISMA (2020 guidelines. A total of 488 records were initially identified through three major electronic databases: PubMed (n = 190), Scopus (n = 165), and Embase (n = 133). After removing 71 duplicate records, 417 unique records were screened based on titles and abstracts. Of these, 209 records were excluded for not meeting the inclusion criteria. The full texts of 208 articles were sought for retrieval; however, 41 could not be accessed. Subsequently, 167 articles were assessed for eligibility through a detailed review. During this phase, 35 narrative reviews, 28 editorials and letters, 25 conference abstracts, 22 pediatric studies, 21 animal studies, 21 studies without adjusted effect estimates, and eight duplicates or irretrievable full texts were excluded. Ultimately, eight studies met the eligibility criteria and were included in the final systematic review. This process ensured a rigorous selection of high-quality observational data relevant to assessing the long-term adverse effects of PPI use.

Characteristics of the Selected Studies

Table 1 presents the characteristics of the eight selected studies assessing the long-term effects of PPI use. Most studies, including cohorts and meta-analyses, found significant associations between prolonged PPI use and adverse outcomes such as dementia, CKD, fractures, and vitamin B12 deficiency. Effect estimates were generally adjusted for confounders. One study also noted that many long-term users lacked clear indications for PPI therapy, raising concerns about potential overuse.

**Table 1:** Summary of the characteristics of the included studies. PPI: proton pump inhibitor; CKD: chronic kidney disease; FA: fractional anisotropy; MD: mean diffusivity; ARIC: atherosclerosis risk in communities; ALA: acid-lowering agent; H₂RA: histamine-2 receptor antagonist; OR: odds ratio; HR: hazard ratio; RR: relative risk; CI: confidence interval; GP: general practitioner

First author, year	Study type	Population/Sample size	Antacid type and duration	Outcome(s) assessed	Main findings	Effect estimates (OR, HR, RR)	Confounders adjusted
Gomm et al., 2016 [13]	Prospective cohort	73,679 elderly individuals (≥75 years), free of dementia at baseline	Regular PPI use (omeprazole, pantoprazole, lansoprazole, esomeprazole, rabeprazole); duration not specified	Incident dementia	Regular PPI users had a significantly higher risk of developing dementia compared to non-users	HR: 1.44 (95% CI: 1.36–1.52)	Age, sex, comorbidities, polypharmacy
Wu et al., 2023 [14]	Systematic review and meta-analysis	6,829,905 participants from 10 observational studies	PPIs; duration varied across studies	CKD	PPI use is significantly associated with an increased risk of CKD compared to non-use	RR: 1.72 (95% CI: 1.02–2.87)	Yes – adjusted in the original observational studies included
Alaeddin et al., 2024 [15]	Cross-sectional study	7,465 participants (Rhineland Study)	PPIs; short term (<3 years) and long term (≥3 years)	Cognitive performance; brain macrostructure and microstructure (FA, MD)	Long-term PPI use, especially in younger adults, is associated with poorer cognition and higher MD in cognitive-related regions, suggesting white matter disruption	Not reported as OR/HR/RR – used β-coefficients (linear regression)	Yes – Multivariate linear regression adjusting for multiple confounders
Northuis et al., 2023 [16]	Prospective cohort	5,712 dementia-free participants (ARIC Study)	PPIs; duration categorized as 0 days, 1 day–2.8 years, 2.8–4.4 years, >4.4 years	Incident dementia	Cumulative PPI use >4.4 years was associated with a 33% increased risk of dementia. No significant risk with shorter durations or current use at baseline	HR: 1.3 (95% CI: 1.0–1.8) for >4.4 yrs use	Yes – demographics, comorbidities, other medication use
Jung et al., 2015 [17]	Systematic review and meta-analysis	Four case-control studies (4,254 cases, 19,228 controls) + one observational study	Acid-lowering agents (ALAs) (long-term use; duration not specified)	Vitamin B12 deficiency	Long-term ALA use was significantly associated with vitamin B12 deficiency.	HR: 1.83 (95% CI: 1.36–2.46), p = 0.00	Not clearly stated (varied by study)
Poly et al., 2019 [18]	Meta-analysis of observational studies	24 studies; 2,103,800 participants (319,568 hip fracture cases)	PPIs (short- and long-term use, ≥1 year)	Hip fracture	PPI use was significantly associated with increased hip fracture risk. Risk increased with higher doses and longer duration. No similar association was seen with H_2_RA use	RR: 1.20 (95% CI: 1.14–1.28), p < 0.0001 (overall); RR 1.24 (95% CI: 1.10–1.40) for long-term use	Yes (varied across included observational studies)
Eom et al., 2011 [19]	Meta-analysis of observational studies	11 studies (5 case-control, 3 nested case-control, 3 cohort)	PPIs and H₂RAs; focus on long-term use	Any fracture and hip fracture	PPI use was associated with increased fracture risk, especially with long-term use. H₂RA use was not significantly associated with fracture risk	PPI: OR: 1.29 (95% CI: 1.18–1.41); long-term PPI: OR: 1.30 (CI: 1.15–1.48); hip fracture: OR: 1.34 (CI: 1.09–1.66); H₂RA: OR: 1.10 (CI: 0.99–1.23)	Yes (in individual studies as reported)
Reimer & Bytzer, 2009 [20]	Cross-sectional analysis	42,634 patients from 22 GPs; 901 long-term PPI users analyzed	PPI use ≥120 tablets/year (long-term)	Indication for PPI use; patient characteristics	Only 27% of long-term users had verified indications; 71% without verified indications reported reflux symptoms. 61% had attempted withdrawal, yet most continued daily use	Not applicable	No (descriptive study)

Quality Assessment

As shown in Table 2, most studies were rated as having a moderate risk of bias, primarily due to the observational design, potential selection bias, and limitations in establishing causality. Only one study demonstrated low risk, supported by strong methodology and confounder adjustment. The systematic reviews generally scored moderate to high due to concerns regarding the quality of included primary studies.

**Table 2:** Risk of bias assessment of included studies.

First author, year	Study type	Risk of bias tool applied	Risk of bias judgment	Comments
Gomm et al., 2016 [13]	Prospective cohort	Newcastle-Ottawa Scale	Moderate	Good selection and outcome assessment, but potential residual confounding due to the observational nature
Wu et al., 2023 [14]	Systematic review and meta-analysis	AMSTAR 2	High	Included only observational studies; risk of bias in primary studies not adequately addressed
Alaeddin et al., 2024 [15]	Cross-sectional study	AXIS Tool	Moderate	Adequate methodology, but potential for selection bias and temporality cannot be inferred
Northuis et al., 2023 [16]	Prospective cohort	Newcastle-Ottawa Scale	Low	Strong cohort design with time-varying exposure and robust confounder adjustment
Jung et al., 2015 [17]	Systematic review and meta-analysis	AMSTAR 2	Moderate	Generally sound, but limited to case-control studies, which introduces inherent bias
Poly et al., 2019 [18]	Meta-analysis of observational studies	AMSTAR 2	Moderate	Risk of bias due to the observational nature of included studies, though the large sample size supports findings.
Eom et al., 2011 [19]	Meta-analysis of observational studies	AMSTAR 2	Moderate	Reasonably comprehensive, but some included studies had high heterogeneity and inconsistent adjustment
Reimer & Bytzer, 2009 [20]	Cross-sectional	AXIS tool	Moderate	Large sample, but causality cannot be established, and indication bias may be present

Discussion

The majority of studies in this review suggest a significant association between long-term PPI use and adverse health outcomes, including increased risks of dementia, CKD, vitamin B12 deficiency, and bone fractures. For instance, Gomm et al. [13] and Northuis et al. [16] both reported a higher risk of incident dementia among long-term PPI users, with hazard ratios of 1.44 and 1.3, respectively, indicating consistent findings across large cohort studies. Similarly, Wu et al. [14] and Poly et al. [18] showed a notable link between PPI use and CKD or hip fractures, respectively, reinforcing the potential systemic effects of prolonged acid suppression therapy. While most meta-analyses and observational studies reported statistically significant outcomes, variability in duration definitions and confounder adjustments was evident. Among the high-quality studies, those with larger sample sizes and robust multivariate adjustments, such as the ARIC and Rhineland cohorts, had a substantial influence on the review’s overall conclusions.

Beyond the statistical associations, several mechanisms help contextualize these findings. Long-term PPI use has been shown to impair the absorption of essential nutrients such as magnesium and vitamin B12, which are critical for cognitive function and bone health. Studies such as Alaeddin et al. [15] provided neuroimaging-based evidence of microstructural brain changes associated with prolonged PPI exposure, potentially implicating white matter disruption as a pathway for cognitive impairment. Furthermore, the duration of use appears to be a pivotal factor, as shorter or intermittent PPI therapy was not consistently linked to adverse outcomes. Distinctions also emerged when comparing PPIs with H2 receptor antagonists, as the latter generally showed weaker or no associations with negative outcomes, as seen in studies by Eom et al. [19] and Poly et al. [18]. This suggests that the pharmacodynamic intensity of acid suppression may play a role in mediating long-term risks. Overall, the findings underscore the importance of cautious prescribing and routine re-evaluation of chronic PPI therapy, particularly in older adults or those at higher baseline risk for the studied outcomes.

The findings of this review align closely with previously published meta-analyses that have highlighted potential risks linked to long-term PPI use, particularly concerning dementia, CKD, and micronutrient deficiencies. For instance, the meta-analysis by Jung et al. [17] reinforced concerns about vitamin B12 deficiency due to acid suppression, while others, such as Gomm et al. [13], linked PPIs with an increased risk of dementia. These patterns are consistent with recent cautionary positions adopted by clinical societies such as the American Gastroenterological Association (AGA), which have underscored the need to curtail unnecessary long-term PPI use [21,22]. The AGA and other professional bodies have repeatedly emphasized rational PPI prescribing, particularly because a large proportion of chronic users lack verified indications, a trend also demonstrated in studies such as Reimer et al. [20].

Despite the coherence of findings, several limitations temper the strength of the conclusions drawn from the included studies. A major concern is that most of the evidence stems from observational or retrospective analyses, which inherently restrict the ability to establish causality. Moreover, patients prescribed PPIs for extended durations often have underlying comorbidities such as GERD, chronic nonsteroidal anti-inflammatory drug use, or cardiovascular conditions, all of which may independently predispose them to adverse outcomes, introducing potential confounding. While most studies adjusted for age and some clinical variables, residual confounding cannot be ruled out. Additionally, there may be a degree of publication bias, as studies reporting significant harms are more likely to be published, potentially exaggerating the overall risk profile of PPIs.

From a clinical and public health perspective, these findings underline the importance of re-evaluating the widespread and often indefinite use of PPIs, especially in low-risk individuals or those lacking clear therapeutic indications [23]. Healthcare providers should regularly reassess the necessity of ongoing PPI therapy and explore opportunities for step-down approaches or discontinuation. The implementation of deprescribing protocols and integration of shared decision-making between clinicians and patients can help minimize unnecessary exposure while preserving therapeutic benefit for those who truly need acid suppression [24,25]. Education initiatives targeting both prescribers and patients could further encourage judicious use and improve long-term outcomes.

Given the limitations of existing studies, future research should prioritize randomized controlled trials or well-designed prospective cohort studies that adjust more rigorously for baseline comorbidities and medication use. Studies should also incorporate objective biomarkers such as bone mineral density, serum vitamin B12 levels, cognitive function testing, and renal filtration markers to better elucidate causal pathways. Long-term safety registries tracking real-world PPI users over extended periods could help validate and refine current evidence, ultimately guiding safer clinical use. There remains a significant gap in understanding the differential risk profiles of various PPI types and dosages, which future work should also address.

## Conclusions

While accumulating data suggest potential associations between chronic PPI use and adverse health outcomes, these must be interpreted with caution and in the clinical context. PPIs remain among the most effective and well-tolerated treatments for acid-related disorders, and their benefits are clear when used appropriately. Clinicians should be mindful of outcome-specific risks: for dementia, caution is warranted in elderly patients requiring prolonged therapy, with periodic cognitive assessments; for CKD, renal function should be monitored in long-term users, particularly those with baseline comorbidities; and for fractures, bone health and calcium/vitamin D status should be considered, especially in postmenopausal women or other high-risk groups. The emphasis, therefore, should be on ensuring PPI use is evidence-based and regularly reviewed. A balanced approach that avoids both underuse in appropriate cases and overuse in low-risk individuals is essential for maximizing therapeutic benefit while minimizing harm.
